# Exploring the Therapeutic Potential of *Theobroma cacao* L.: Insights from In Vitro, In Vivo, and Nanoparticle Studies on Anti-Inflammatory and Anticancer Effects

**DOI:** 10.3390/antiox13111376

**Published:** 2024-11-11

**Authors:** Przemysław Sitarek, Anna Merecz-Sadowska, Joanna Sikora, Weronika Osicka, Igor Śpiewak, Laurent Picot, Tomasz Kowalczyk

**Affiliations:** 1 Department of Medical Biology, Medical University of Lodz, Muszynskiego 1, 90-151 Lodz, Poland; 2 Department of Allergology and Respiratory Rehabilitation, Medical University of Lodz, 90-725 Lodz, Poland; anna.merecz-sadowska@uni.lodz.pl; 3 Department of Bioinorganic Chemistry, Medical University of Lodz, Muszynskiego 1, 90-151 Lodz, Poland; joanna.sikora@umed.lodz.pl; 4 Students Research Group, Department of Medical Biology, Medical University of Lodz, 90-151 Lodz, Poland; weronika.osicka@stud.umed.lodz.pl; 5 Students Research Group, Department of Molecular Biotechnology and Genetics, Faculty of Biology and Environmental Protection, University of Lodz, Banacha 12/16, 90-237 Lodz, Poland; igor.spiewak@edu.uni.lodz.pl; 6 Littoral Environnement et Sociétés UMRi CNRS 7266 LIENSs, La Rochelle Université, 17042 La Rochelle, France; laurent.picot@univ-lr.fr

**Keywords:** *Theobroma cacao*, anticancer, anti-inflammatory, polyphenols, in vitro studies, in vivo studies, nanoparticles

## Abstract

*Theobroma cacao* L., commonly known as cocoa, has been an integral part of human culture and diet for thousands of years. However, recent scientific research has highlighted its potential therapeutic properties, particularly in the treatment of cancer and inflammatory diseases. This comprehensive review analyzes the anti-inflammatory and anticancer effects of *Theobroma cacao* extracts combined with nanoparticles using in vitro and in vivo studies. Its diverse biological activity can be attributed to its rich phytochemical profile, including polyphenols, flavonoids, and alkaloids. In vitro studies have found that cocoa extracts, alone or in combination with nanoparticles, inhibit cancer cell proliferation, induce apoptosis and modulate key signaling pathways in various cancer cell lines. The extracts have also been found to reduce tumor growth and enhance the efficacy of conventional chemotherapeutic agents, potentially reducing their side effects, in vivo. Its anti-inflammatory properties are based on its ability to modulate inflammatory mediators, inhibit NF-κB signaling, and regulate macrophage polarization. These effects have been observed in both cellular and animal models of inflammation. This review opens up new possibilities for future research and therapeutic applications, highlighting the potential of *Theobroma cacao* as a valuable complementary approach in the treatment and prevention of cancer and inflammatory diseases.

## 1. Introduction

*Theobroma cacao* L., the botanical name for the cacao tree, means “food of the gods”, a fitting title for a plant that has played a significant role in human history for thousands of years. The history of cacao is rich and multifaceted, beginning with the ancient Mesoamerican civilizations and extending to its global prominence today. This paper explores the cultural significance and historical use of *T. cacao*, from its origins, through its introduction to Europe, and ultimately to its worldwide impact. Cacao trees originally grew in the river valleys of South America, and by the seventh century AD, the Mayan Indians had introduced them to Mexico. They were also domesticated and cultivated by other Central American Indian groups, including the Aztecs and the Toltecs. The word chocolate comes from the Aztec language, derived from inter alia xhocolatl or cacahuatl. Ancient texts describe various cacao mixtures used for ceremonial, medicinal, and culinary purposes [1,2,3]. The name *Theobroma cacao* was coined by the Swedish botanist Carl Linnaeus in 1753 and published in *Species Plantarum*. *Theobroma* itself is derived from the Greek *broma*, meaning food, and Theo, meaning god, which translates as food of the gods [4,5,6]. The cacao tree thrives at elevations of 200–400 m in regions receiving 1000–3000 mm of annual rainfall. It needs a humid environment with consistent rainfall and rich soil. *Theobroma cacao* can grow only within 10 degrees north or south of the equator, favoring the moist, shady regions of tropical rainforests [7,8]. One of the greatest individual causes of death is cancer, caused by harmful chemicals, viruses, agents that produce free radicals, certain environmental toxins, and unavoidable everyday factors. Traditional cancer treatments such as chemotherapy, radiotherapy, and surgery typically provide only limited and temporary relief. Therefore, there is great interest in identifying alternative remedies for cancer that are widely accessible, cost-effective, and have minimal side effects [9,10,11,12]. *Theobroma cacao* appears to have anticancer and anti-inflammatory properties thanks to its flavonoid, polyphenol, and alkaloid content [13,14,15]. Cocoa also has cardioprotective, neuroprotective, and hepatoprotective effects, and cocoa leaf and bark extracts have demonstrated protective effects against doxorubicin-induced oxidative stress and organ damage [16,17,18,19,20]. Among promising strategies to enhance the therapeutic potential of *T. cacao* extracts, nanoparticle-based delivery systems have emerged as a particularly interesting approach. Nanoparticles can improve the bioavailability of bioactive compounds, enable targeted delivery to specific tissues, and potentially enhance the therapeutic efficacy of cocoa extracts. The combination of *T. cacao* extracts with nanoparticles represents an innovative direction in developing more effective treatments for cancer and inflammatory diseases [21].

The aim of this study is to comprehensively evaluate the anti-inflammatory and anticancer properties of *T. cacao* extract in vitro and in vivo, alone and in combination with nanoparticles. This research aims to elucidate the potential mechanisms underlying these effects and explore the potential of *T. cacao* as a source of novel bioactive therapeutic compounds for the treatment of cancer and inflammatory diseases.

## 2. Experimental Paper Selection Criteria

This review focuses on in vitro and in vivo studies of plant extracts from *Theobroma cacao* and the potential mechanisms of their anticancer and anti-inflammatory effects. The studies were identified using the PubMed, Google Scholar, and Scopus databases and included data published between 2004 and 2024. The following key words were used in the search: *Theobroma cacao* extract, secondary metabolite, in vitro study, in vivo study, anticancer potential of *Theobroma cacao*, anti-inflammatory potential of *Theobroma cacao*, anticancer potential of *Theobroma cacao* in combination with nanoparticles, anti-inflammatory potential of *Theobroma cacao* in combination with nanoparticles, bioactive compounds in *Theobroma cacao*, therapeutic potential of *Theobroma cacao*, synergistic anticancer effects of Theobroma cacao and nanoparticles. The articles published in languages other than English or abstracts were excluded. Duplicate articles from different databases were also excluded. All the inclusion and exclusion criteria were checked again after these articles had been removed. Finally, after searching the above sources, 203 articles were found, including the following: anticancer in vitro effect of *Theobroma cacao* extract (55 articles ), in vitro anti-inflammatory effect of *Theobroma cacao* extract (49 articles), in vivo anticancer effect of *Theobroma cacao* extract (31 articles), in vivo anti-inflammatory effect of *Theobroma cacao* extract (46 articles), biological effect of nanoparticles with *Theobroma cacao* extract (22 articles). Due to similarities, lack of relevance to the topic, and imprecise information contained within them, 98 records were excluded. As a result, the final analysis includes 105 articles in total, of which 50 are presented in tables.

Each selected scientific paper was analyzed, and the following data were selected and tabulated: scientific name of the plant, plant parts used to prepare the extract or isolate the pure compound, type of extract, class of compounds or different compounds identified in the extract, cancer cell lines used or animal model/cell line inoculated with carcinogenic compounds, activity or mechanism of action, and reference. Articles explaining the mechanism(s) of action of plant extracts derived from *T. cacao* are discussed in the main text before being presented in tables.

## 3. Agricultural Livelihoods and Socio-Economic Development

**Figure 1:**
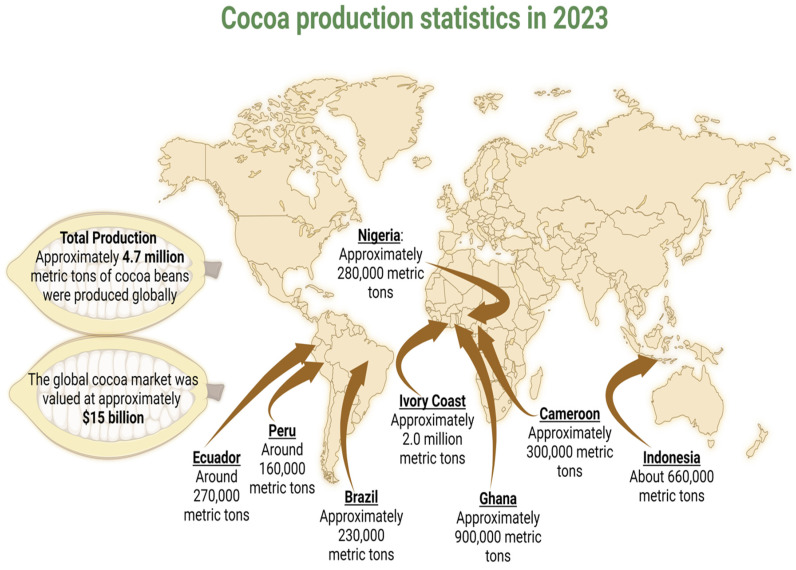
Global cocoa production.

The global demand for chocolate and cocoa products drives a robust international market for cacao beans. The chocolate industry is a multi-billion dollar market, with annual global chocolate sales exceeding $100 billion [22,23]. Cocoa cultivation is an important source of income for millions of smallholder farmers in tropical regions, mainly in West Africa, Latin America, and Southeast Asia. Leading producers, including Ivory Coast, Ghana, Indonesia, and Brazil, rely heavily on cocoa cultivation as a key component of their agricultural sectors [24] (Figure 1). In these countries, cocoa exports are crucial, accounting for a significant proportion of their gross domestic product (GDP) and export earnings. For smallholder farmers, cocoa is a stable cash crop that provides an essential income. Cocoa cultivation also has cultural significance in many production regions. It supports traditional farming practices and contributes to the preservation of local heritage. In some areas, cocoa cooperatives play a key role in community organization, empowering farmers through collective bargaining and shared resources. The economic stability provided by cocoa cultivation helps to improve living standards, fund education, and provide health care. In addition, cocoa supports ancillary industries such as local processing, transport, and agricultural supply, further stimulating the rural economy.

## 4. *Theobroma cacao* L.—Characteristics, Secondary Metabolites, and Health Benefits

**Figure 2:**
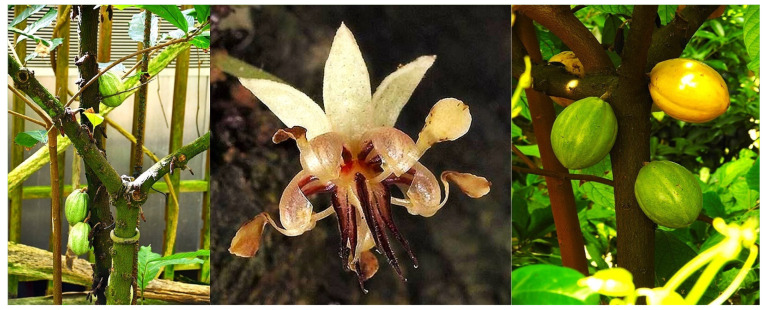
*Theobroma cacao* L. (courtesy of the Missouri Botanical Garden and the Royal Botanic Gardens, Kew, ©RBG Kew).

The *Theobroma* genus (*Theobroma cacao* L.), globally renowned as the basis of chocolate (Figure 2), lies within the family Malvaceae, a large and diverse family of flowering plants. It includes around 244 genera and over 4225 species, encompassing a variety of growth forms, such as trees, shrubs, and herbaceous plants. Its members are distributed worldwide, particularly in tropical and subtropical regions. Among these, *Theobroma cacao*, the cocoa tree, is a particularly desirable crop for its seeds [25]. The cocoa tree thrives exclusively in humid, tropical regions, and cocoa has become a vital agricultural product in numerous developing nations across West Africa, where it accounts for over 70% of global production, as well as in Southeast Asia, Latin America, and the Caribbean [26]. The seeds of *T. cacao*, commonly enjoyed in cocoa drinks and chocolate, are recognized as a rich source of bioactive compounds, particularly polyphenols, alkaloids phytosterols, and fatty acids; these are believed to confer many of the proposed protective effects associated with the consumption of cocoa and chocolate. These secondary metabolites not only contribute to the unique flavor profile of cocoa but also play a significant role in its potential health benefits [9,10].

*Theobroma cacao* is particularly rich in polyphenols, a diverse group of plant-based compounds. Cocoa beans contain three main groups of polyphenols: catechins (about 37% of the polyphenol content), anthocyanidins (around 4%), and proanthocyanidins (approximately 58%). The most abundant catechin is (−)-epicatechin, which makes up as much as 35% of the total catechin content, with smaller amounts of (+)-catechin, (+)-gallocatechin, and (−)-epigallocatechin. For anthocyanidins, the primary compounds are cyanidin-3-α-L-arabinoside, and cyanidin-3-β-D-galactoside. In the case of procyanidins, the main components are dimers, trimers, or oligomers of flavan-3,4-diol connected by 4 → 8 or 4 → 6 bonds, with the most significant procyanidins being B1, B2, B3, B4, B5, C1, and D [27,28]. Another group of compounds present in *T. cacao* are alkaloids. Among all structurally-related purine alkaloids found in chocolate (methylxanthines), theobromine stands out as the primary compound. *Theobroma cacao* has two major classes of phenylpropanoids, viz. cinnamic acid and tocopherol derivatives [29]. Jalal and Collin isolated p-coumaric acid and caffeic acid, two common cinnamic acid derivatives, along with p-coumaroylquinic acid and chlorogenic acid, i.e., cinnamic acid derivatives containing quinic acid, from *T. cacao* leaves [30]. The same compounds were detected by UHPLC-MS/MS study in the leaves, seeds, and pericarp of *Theobroma* genus by Tauchen et al. [31].

**Figure 3:**
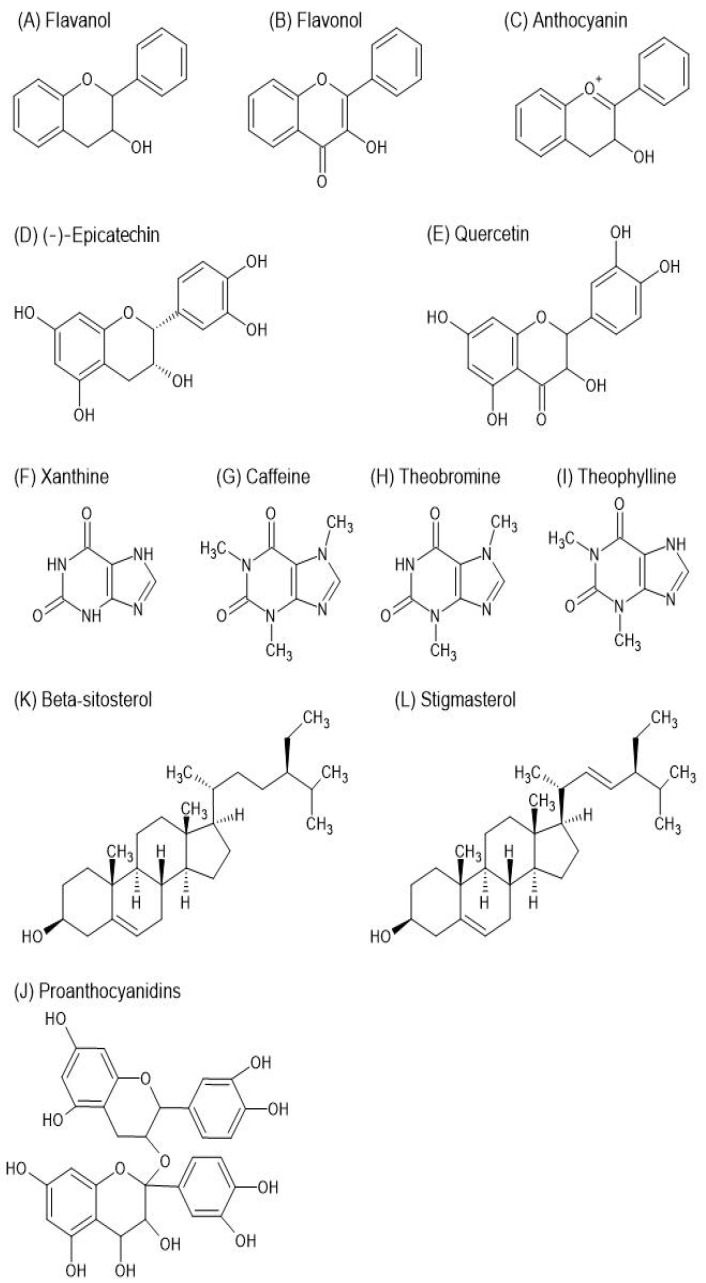
Chemical structure of selected secondary metabolites of *Theobroma cacao* L. (ChemSketch (freeware), version 2023.2.4, Advanced Chemistry Development, Inc. (ACD/Labs), Toronto, ON, Canada, www.acdlabs.com accessed on 23 June 2024).

The seeds of *Theobroma cacao*, also known as cocoa beans, are rich in fats. Approximately 50–57% of the dry weight of whole cocoa beans consists of lipids, commonly referred to as cocoa butter. This cocoa butter, predominantly found in dark chocolate, typically consists of around 33% oleic acid, 25% palmitic acid, and 33% stearic acid [32]. *Theobroma cacao* contains a wealth of minerals, including potassium, phosphorus, copper, iron, zinc, and magnesium. These minerals play an important role in the proper functioning of the body and are involved in most physiological processes, e.g., maintaining the acid–base balance, transporting some substances, and maintaining healthy bones and teeth. Therefore *T. cacao*, in the form of high-cocoa chocolate rich in nutrients, can be included in a well-rounded diet, when consumed in moderation [33]. Selected secondary metabolites contained in *Theobroma cacao* are shown in Figure 3.

The bioactive compounds in *Theobroma cacao* have been associated with various potential health benefits, including antioxidant properties, metabolic effects, anti-inflammatory potential, and neuroprotective effects, as well as cardiovascular health and anticancer properties [10].

Polyphenols and flavonoids exhibit strong antioxidant properties, acting as free radical scavengers and potentially reducing oxidative stress in the body [34]. Studies have also shown cocoa consumption to have promising metabolic effects. Cocoa has been demonstrated to alleviate obesity. Studies showed that mice fed a high-fat diet supplemented with cocoa demonstrated less weight gain, attenuated insulin resistance, and reduced severity of obesity-related fatty liver disease. Moreover, it has been proven that daily consumption of 40 g/d of dark chocolate rich in cocoa lowers markers of oxidative stress, hepatocyte apoptosis and ALT in the serum compared to the consumption of milk chocolate [35]. Furthermore, cocoa and its procyanidins demonstrate significant anti-inflammatory potential. They may provide anti-inflammatory benefits by modulating cytokine production and regulating NF-κB target gene expression, which could have implications for various inflammatory conditions [36]. Notably, in vitro studies have suggested that cocoa polyphenolic extracts may have neuroprotective effects, which may be significant in neurodegenerative conditions. For example, in Alzheimer’s disease, cocoa polyphenolic extracts not only exhibit antioxidant properties but also provide neuroprotective effects. These neuroprotective effects are linked to the activation of the BDNF survival pathway in cells treated with Aβ plaques or Aβ oligomers, resulting in a decrease in neurite dystrophy. Alzheimer’s disease is characterized by an increased production of amyloid (A)β oligomers, which activates microglia, leading to the release of inflammatory substances and neuronal death [37]. Cocoa extracts have demonstrated cardioprotective potential by exerting hypoglycemic and hypocholesterolemic effects, with treatment lowering serum glucose levels and improving lipid profiles. The consumption of chocolate and chocolate-containing confections has been found to induce relatively low levels of postprandial glycemia compared to equivalent amounts of carbohydrates in starchy foods such as bread, rice, and potatoes. They may help lower serum glucose levels and improve lipid profiles, potentially contributing to the prevention of cardiovascular diseases and diabetes [38]. Additionally, the phytosterols present in cocoa seeds lower total plasma cholesterol and low-density lipoprotein levels by inhibiting cholesterol absorption in the intestine. Cocoa seeds contain 2–3 mg of plant sterols per gram of fat, mainly β-sitosterol and stigmasterol. The fatty acid profile of cocoa may also contribute to its cardiovascular benefits, comprising primarily triacylglycerols (TAGs) with 2-oleyl glycerides (O) of palmitic (P) and stearic (S) acids (POP, POS, SOS) [39].

Polyphenols are especially renowned for their beneficial impact on cancer. For example, they appear to prevent the progression of cancer and have been found to induce non-apoptotic cell death and block the cell cycle in the G2/M phase, enhancing their antiproliferative properties [28]. In addition, in laboratory studies, cocoa compounds such as epicatechin, catechin, quercetin, and extracts of procyanidins and B-type dimeric procyanidins have been found to decrease the expression or activity of NF-κB and AP-1 in Hodgkin and Reed–Sternberg (HRS), Daudi, human acute monocytic leukemia (THP-1), and Jurkat cancer cell lines. Pentameric procyanidin from *T. cacao* was found to inhibit breast cancer cell growth by inducing mitochondrial effects that lead to growth arrest or trigger apoptotic and non-apoptotic cell death processes. Additionally, research on the novel mitochondriotoxic molecule F16 has shown that compounds causing mitochondrial depolarization can inhibit breast cancer cell growth through G1 arrest, apoptosis, or necrosis, depending on the genetic makeup of the cells [10].

These diverse potential health benefits highlight the growing interest in *Theobroma cacao* as a possible source of nutraceuticals and functional food components. As research in this area continues to expand, it is becoming increasingly clear that the use of *Theobroma cacao* may extend far beyond chocolate production, opening new avenues for health promotion and disease prevention.

## 5. Anticancer and Anti-Inflammatory Effects of *Theobroma cacao* Extract In Vitro and In Vivo

The relationship between inflammation and cancer has been the subject of growing interest in recent years. Chronic inflammation can contribute significantly to the development and progression of cancer through multiple mechanisms. Inflammatory cells, particularly tumor-associated macrophages (TAMs), can promote tumor growth by producing growth factors, cytokines, and chemokines that stimulate cell proliferation, angiogenesis, and metastasis [40,41,42]. These cells also generate reactive oxygen and nitrogen species, which can induce DNA damage and genomic instability in proliferating cells. Furthermore, the inflammatory microenvironment can facilitate the survival of neoplastic cells by activating transcription factors such as NF-κB and STAT3, which regulate pro-survival genes [43,44]. Chronic inflammation associated with infections, autoimmune diseases, or environmental factors can create a tumorigenic microenvironment that fosters neoplastic transformation and progression. Understanding this intricate relationship between inflammation and cancer provides valuable insights into potential therapeutic targets and underscores the importance of anti-inflammatory strategies in cancer prevention and treatment [45,46,47]. *Theobroma cacao* extracts exhibit diverse anticancer effects through multiple mechanisms targeting various aspects of cancer cell biology. These extracts, rich in bioactive compounds, demonstrate antiproliferative, pro-apoptotic, and cell cycle regulatory activities across different cancer cell lines. The antiproliferative effects of cocoa extracts are evidenced by their ability to inhibit cancer cell growth in various cell lines, including MCF-7 (breast cancer), HepG2 (liver cancer), and Caco-2 (colon cancer) [34,48]. This growth inhibition is often associated with cell cycle arrest, particularly at the G2/M phase, as observed in Caco-2 cells [49]. The extracts also modulate key enzymes involved in polyamine biosynthesis, such as ornithine decarboxylase and S-adenosylmethionine decarboxylase, potentially contributing to their antiproliferative effects [49]. Cocoa extracts induce apoptosis in cancer cells through multiple pathways. For instance, methanolic leaf extracts have been shown to upregulate pro-apoptotic genes (DDIT3, HRK, GADD45G) and increase the activity of caspases 8 and 9 in MCF-7 cells [50]. Additionally, procyanidin-rich extracts can increase intracellular reactive oxygen species (ROS) levels and promote caspase-3-dependent cell death in ovarian cancer cells [51]. Cocoa extracts may also possess antimetastatic properties. For example, procyanidin-rich extracts have been observed to downregulate matrix metalloprotease-2 (MMP2), an enzyme associated with cancer metastasis [51]. Theobromine, a major alkaloid in cocoa, demonstrates significant anticancer potential. It enhances the cytotoxicity of doxorubicin in LoVo colon cancer cells and modulates proteins associated with proliferative and anti-apoptotic pathways, including PDE4, ERK, NF-κB, and Akt/mTOR [34,52]. Theobromine also activates pro-apoptotic pathways involving JNK and p38-MAPK [52]. It is important to note that the anticancer effects of cocoa extracts can vary depending on the plant part used, extraction method, and specific composition of bioactive compounds. Furthermore, the relationship between antioxidant activity, total phenolic content, and anticancer effects is complex and does not always show a direct correlation [53]. Finally, *Theobroma cacao* extracts exhibit multifaceted anticancer activities, including antiproliferative, pro-apoptotic, and potentially antimetastatic effects. These activities are mediated through various molecular mechanisms, highlighting the potential of cocoa-derived compounds in cancer research and therapy. However, further research, particularly in vivo studies and clinical trials, is needed to fully elucidate the therapeutic potential and mechanisms of action of these extracts. Other studies are presented in Table 1 below.

**Table 1:** Anticancer in vitro effect of *Theobroma cacao* extract.

Part of the Plant	Class of Compounds	Cell Line	Ic_50_	Activity/Mechanism/Effects	Ref.
Cocoa beans	phenylpropenyl-amino acids, hydroxycinnamic-amino acid conjugates, procyanidin compounds, fatty acids and lysophospholipids	MCF-7, Hep-G2, OE19, Caco-2	Indonesian cocoa beans: MCF-7—254.20 µg/mL Hep-G2—122.00 µg/mL OE19—903.30 µg/mL Caco-2—104.90 µg/mL Peruvian cocoa beans: MCF-7—708.30 µg/mL Hep-G2—199.70 µg/mL OE19—>1000 µg/mL Caco-2—133.90 µg/mL	The extract did not significantly inhibit proliferation of all cancer cell lines.	[48]
Cocoa leaf extract	-	MCF-7	41.43 µg/mL	The extract did not significantly inhibit proliferation of all cancer cell lines.	[34]
Cacao fruit powder	polyphenols flavonoids	HeLa, CaCo2	HeLa: 1810 µg/mL, 2170 µg/mL CaCo2: 2650 µg/mL	The extract inhibited proliferation of both cancer cell lines.	[54]
Theobromine extract	theobromine	LoVo, LoVo/Dx	-	Theobromine highly enhanced cytotoxic and resistance reversal potency of MAE-TPR.	[55]
Cocoa bean phenolic extract	protocatechuic acid, p-hydroxybenzoic acid, ideain, catechin, chlorogenic acid, caffeic acid, epicatechin, cyanidin, quercetin, kaempferol, procyanidin B1, procyanidin B2, clovamide	AML12, MLP29	-	The cocoa extract inhibited drug-triggered cytotoxicity in liver, possibly by activating autophagy. The phenolic compounds protected the cells from celecoxib-induced viability inhibition. Apoptotic pathways (e.g., Bax) are the main target for cocoa extract.	[50]
Edel cocoa bean extract from fermented and unfermented cocoa beans	polyphenols	Human gingival fibroblast cells	1552.877 µg/mL (from fermented cocoa extract) 32.1282 µg/mL (from unfermented cocoa extract)	The extract from fermented cocoa beans did not show a cytotoxic effect, but the extract from unfermented beans showed a cytotoxic effect on fibroblast cells.	[49]
Cocoa leaf extract	methanolic extract	MCF-7	6.4 µg/mL (concentration after 48 h)	The compounds present in the methanolic leaf extract induced apoptosis in breast cancer cells by inducing cell shrinkage and membrane blebbing. The bioactive fraction upregulated pro-apoptotic genes (DDIT3, HRK, GADD45G) and increased the activity of caspase 3, caspase 8, and caspase 9.	[56]
Cocoa powder	caffeine, theobromine, flavonols, procyanidins	Caco-2	-	The cocoa extract had an anti-proliferative effect by blocking cells at the G2/M phase. Cocoa extract causes polyamine biosynthesis inhibition.	[52]
Cocoa leaf, bark, husk, unfermented cocoa shell, fermented cocoa shell, root, cherelle, pith extracts	methanolic extract	MCF-7, A549, HeLa, HepG2, HT-29, MDA-MB-231, WRL-68	For MCF-7 cell line: Leaf—41.43 µg/mL Bark—71.97 µg/mL Husk—62.23 µg/mL Unfermented shell—65.03 µg/mL Fermented shell—242.33 µg/mL Pith—329.67 µg/mL Root—76.40 µg/mL Cherelle—68.90 µg/mL	The cocoa leaf extract had strong cytotoxic activity against the MCF-7 cell line. The hexane-partitioned fraction had the highest cytotoxic effect.	[57]
Theobromine extract	theobromine	U87-MG	-	Theobromine showed antiproliferative properties on the U87-MG cell line. This activity was mediated by the modulation of proteins associated with proliferative and anti-apoptotic pathways (e.g., PDE4, ERK, NF-κB, Akt/mTOR) and the activation of the pro-apoptotic pathway by JNK, p38-MAPK.	[58]
Phenolic cocoa powder extract	theobromine, procyanidin B1, procyanidin B2, catechin, epicatechin	HepG2	-	The cocoa extract protected cells from oxidative stress.	[59]
Roasted cocoa, unroasted cocoa, roasted fermented cocoa, unroasted fermented cocoa,	phenols	A549	-	Cocoa bean extracts inhibited cell proliferation, stopped the cell cycle in different phases, and increased apoptosis process in the A549 cell line.	[53]
Cocoa seeds	albumin, globulin, prolamin, glutelin	L5178Y	Unfermented cocoa: Albumin—3140 (µg protein/mL) Globulin—2890 (µg protein/mL) Glutelin—580 (µg protein/mL) Semi-fermented cocoa: Albumin—1510 (µg protein/mL) Globulin—2210 (µg protein/mL) Glutelin—220 (µg protein/mL)	Antitumor activity was observed only in the albumin fraction that inhibited the growth of lymphoma cells. It may be associated with sulfur and hydrophobic amino acids. Antioxidant activity was observed in the glutelin and albumin fractions. No correlation was found between antitumor and antioxidant activity.	[51]
Cocoa leaf, bark, husk, fermented and unfermented shell, pith, root, cherelle	methanolic extract	MCF-7, MDA-MB-231, HepG2, HT-29, A549, HeLa, WRL-68	41.4 μg/mL–857.04 μg/mL	The root extract had the highest antioxidant activity, but only the cherelle extract inhibited lipid peroxidation. The leaf extract had highest antiproliferative potential. A negative correlation was found between antioxidant activity, total phenolic content, and anticancer effect.	[60]
Procyanidin rich cocoa powder extract	procyanidins, flavan-3-ol, catechin	OAW42, OVCAR3	-	The procyanidin-rich extract increased the intracellular level of ROS. The treatment induced caspase-3-dependent death and the downregulation of MMP2 (a matrix metalloprotease associated with metastasis).	[61]
Cocoa bean husk	polyphenol, flavonoids	PC3, DU145	-	Bean husk includes large amounts of phenolic compounds, which demonstrated antioxidant and anticancer activity on prostate cancer cell lines.	[62]
Cocoa pod husk	methanolic extract, lupeol, syringaresinol, catechol, squalene	MCF-7, HeLa	MCF—7: 161.53 μg/mL, 45.36 μg/mL, 53.91 μg/mL HeLa: 272.58 μg/mL, 82.44 μg/mL, 120.71 μg/mL	The ethyl acetate partition derived from cocoa pod husk had moderate activity against MCF-7 cells and low activity against HeLa. The extract demonstrated high levels of lupeol, syringaresinol, catechol and squalene, which showed anticancer activity.	[63]

The anti-inflammatory properties of *Theobroma cacao* extracts have been extensively studied in various in vitro models, revealing a complex array of mechanisms through which cocoa compounds modulate inflammatory responses. These effects are primarily attributed to the diverse bioactive compounds present in cocoa. Cocoa extracts demonstrate significant modulatory effects on inflammatory mediators. Multiple studies have shown that these extracts can reduce the production of pro-inflammatory cytokines such as TNF-α, IL-1β, and IL-6 in various cell lines, including macrophages and endothelial cells [64,65,66]. This cytokine modulation is often accompanied by a decrease in the expression of their corresponding mRNAs, suggesting regulation at the transcriptional level [64]. The anti-inflammatory action of cocoa extracts also involves the modulation of key signaling pathways. For instance, cocoa polyphenols have been shown to inhibit the nuclear translocation of NF-κB, a crucial transcription factor in inflammatory responses [67]. Additionally, some studies report the activation of MAPK pathways by theobromine, which can lead to complex immune-modulatory effects [68]. Interestingly, cocoa extracts demonstrate the ability to influence macrophage polarization. Phenolic extracts from cocoa beans have been observed to promote a shift from pro-inflammatory M1 macrophages to anti-inflammatory M2 macrophages, thereby altering the overall inflammatory milieu [69]. Moreover, cocoa extracts have demonstrated the ability to maintain gut barrier function and reduce epithelial inflammation [70,71]. Another significant anti-inflammatory mechanism of cocoa extracts is the suppression of nitric oxide (NO) production in activated macrophages [72]. This effect is particularly important given the role of NO in perpetuating inflammatory responses.

Cocoa compounds also show protective effects on cellular structures. Theobromine, for instance, has been reported to protect membrane integrity by reducing levels of inflammatory factors and matrix metalloproteinases [73]. It is worth noting that the anti-inflammatory effects of cocoa extracts can vary depending on the specific composition and preparation method. For example, different fractions of pectin obtained from cocoa pod husks showed varying abilities to modulate macrophage functions and cytokine production [74]. In conclusion, the anti-inflammatory properties of *Theobroma cacao* extracts are mediated through multiple mechanisms, including cytokine modulation, signaling pathway regulation, macrophage polarization, and protection of cellular structures. These diverse effects underscore the potential of cocoa-derived compounds as novel anti-inflammatory agents. However, further research, particularly in vivo studies and clinical trials, is necessary to fully elucidate the therapeutic potential of these extracts in inflammatory conditions. Other studies are presented in Table 2 below.

**Table 2:** In vitro anti-inflammatory effect of *Theobroma cacao* extract.

Part of the Plant	Class of Compounds	Cell Line	Dose	Activity/Mechanism/Effects	Ref.
Cocoa pod husk	Pectin	Mice peritoneal macrophages	25, 50, 100, 200, 400 μg·mL^−1^	Optimized pectin, partially deacetylated pectin, de-esterified pectin, and homogalacturonan pectin obtained from cocoa pod husk are able to modulate some macrophage functions, e.g., the secretion of pro-inflammatory factors (NO, TNF-α, IL-12) and anti-inflammatory IL-10. The optimized pectin fraction showed anti-inflammatory activity, while homogalacturonan pectin increased the number of activated macrophages.	[74]
Theobromine extract	Theobromine	CaCo-2	10–30 μM	Theobromine protected membrane structure integrity by decreasing the level of specific inflammatory factors such as cytokines and matrix metalloproteinases.	[73]
Cocoa powder extract	Flavonoids Polyphenols	NR8383, RAW 264.7	5–100 μg/mL	Cocoa extract lowered the level of TNF-α, IL-1α, IL-6, NO and MCP-1.	[64]
Aqueous cocoa extract	Polyphenols	J744A.1	0.25%, 0.05%	Cocoa extract contains compounds that suppress NO production in macrophages activated by LPS and IFN-γ.	[72]
Dried cocoa beans	Polyphenols	HUVEC	25, 50, 100 ppm	Cocoa extract prevents increases in IL-6 and sVCAM-1 levels in human endothelial cells following induction by plasma from preeclamptic patients.	[65]
“Guiana” cocoa pods	Theobromine, Caffeine, Epicatechin, Procyanidin A1, Procyanidin A2, Procyanidin B2, Procyanidin C1	J774-A1	100 μL	Guiana cocoa shows better inhibition of IL-6 production and stimulation of TNF-α secretion.	[66]
Cocoa beans	Phenolic extract	THP-1	0.1–100 μM	Cocoa extract reduced the inflammatory response in M1 macrophage by increasing the secretion of anti-inflammatory cytokines. It also caused a metabolic switch from the M1 pro-inflammatory to the M2 anti-inflammatory type.	[69]
Cocoa bean shell	Theobromine, Caffeine, Protocatechuic acid, Catechin, Epicatechin, Procyanidin B2, Procyanidin B, Procyanidin A, Quercitin-3-O-glycosides	CaCo-2	10, 25, 50 μg/mL	The extracts inhibited IL-8 and TLR2, and TLR4, indicating that cocoa could interfere with oxysterol-mediated inflammation.	[67]
Theobromine extract	Theobromine	RAW 264.7	1–500 μg/mL	Theobromine activated MAPK and NF-κB signaling pathways, which enhance immune effects. Theobromine increases production of inflammatory factors by p38, JNK, and NF-κB pathways. It also upregulates the expression of iNOS. Higher expression of COX-2 causes increased PGE2 production.	[68]
Procyanidin-rich cocoa extract	Flavanols, Catechin, Epicatechin	CaCo-2, HT-29	Max concetration—100 μg/mL or 10–25 μg/mL	Cocoa extract prevents loss of gut barrier function and epithelial inflammation.	[70]
Cocoa beans	Polyphenols	GMSM-K	31.25, 62.5, 125, 250, 500 μg/mL	Cocoa extract inhibited *F. nucleatum*-induced inflammatory response in monocytes and oral epithelial cells. The extract improves the barrier function of oral epithelial cells.	[71]
Cocoa powder	Aqueous and ethanolic extract	PBMC, THP-1	0.5–10 μg/mL	Cocoa extracts suppressed mitogen-induced degradation of tryptophan. IFN-γ and neopterin production were strongly inhibited by extracts.	[75]
Cocoa extract	-	HUVEC	6.25, 12.5, 25, 50, 100 μg/mL	The cocoa extract inhibited angiotensin-covering enzyme activity and increased the NO level.	[76]

The in vivo anticancer effects of *Theobroma cacao* extracts have been investigated in various animal models, providing crucial insights into their potential therapeutic applications. These studies reveal a complex interplay of mechanisms through which cocoa-derived compounds exert their anticancer activities while also demonstrating protective effects against chemotherapy-induced toxicity. One of the most notable findings is the ability of cocoa extracts to enhance the efficacy of conventional chemotherapeutic agents. For instance, the combination of cocoa extract with doxorubicin has been shown to increase the overall anticancer effect, albeit with a partial increase in systemic toxicity [77]. This synergistic action suggests that cocoa compounds may potentiate the cytotoxic effects of chemotherapy on cancer cells. Furthermore, cocoa powder has demonstrated protective efficacy against vital organ damage induced by doxorubicin, without compromising its chemotherapeutic effect in BALB/c mice with Ehrlich ascites carcinoma [17]. This dual action of organ protection and maintenance of anticancer efficacy is particularly noteworthy, as it addresses one of the major challenges in cancer therapy—reducing side effects while maintaining treatment effectiveness.

The anticancer potential of cocoa extracts is further evidenced by their ability to act as carcinogenic inhibitors. This is achieved through the down-regulation of heat shock protein 90 (Hsp90) and the normalization of heat shock protein expression [78]. Given the role of Hsp90 in stabilizing various oncogenic proteins, its downregulation represents a significant mechanism by which cocoa compounds may inhibit cancer progression. Importantly, cocoa extracts have demonstrated protective effects against chemotherapy-induced organ toxicity. Studies in female BALB/c mice have shown that cocoa extract can protect vital organs such as the heart, liver, and kidney from doxorubicin-induced damage [79]. This protective effect, combined with the synergistic anticancer activity, suggests a potential role for cocoa extracts in improving the therapeutic index of conventional chemotherapy. Moreover, cocoa extracts demonstrated significant antioxidant and hepatoprotective properties in vivo. Studies using BALB/cN mice have shown that cocoa polyphenols favorably modulate cellular redox state and molecular signaling pathways associated with oxidative stress [78]. These extracts have been observed to decrease hepatic cell necrosis and suppress Pkm2, a key enzyme in cancer metabolism. Furthermore, they reduce hepatotoxicity by lowering the activity of serum transaminases and phosphatic phosphatase, indicating a protective effect on liver function [78]. The anticancer effects of cocoa are not limited to direct cytotoxicity or chemotherapy enhancement. Daily consumption of cocoa products has been shown to increase plasma flavonol concentrations in Wistar–Unilever rats, thereby enhancing the overall antioxidant potential of plasma [80]. This systemic increase in antioxidant capacity may contribute to cancer prevention by reducing oxidative stress and DNA damage. In conclusion, the in vivo anticancer effects of *Theobroma cacao* extracts are multifaceted, involving enhancement of chemotherapy efficacy, modulation of oxidative stress and cellular signaling pathways, carcinogenic inhibition, and protection against chemotherapy-induced organ toxicity. These diverse mechanisms highlight the potential of cocoa-derived compounds as adjuvants in cancer therapy, possibly improving treatment outcomes while mitigating side effects. However, further research, including clinical trials, is necessary to fully elucidate the therapeutic potential and safety profile of these extracts in human cancer treatment. Other studies are presented in Table 3 below.

The in vivo anti-inflammatory effects of *T. cacao* extracts have been extensively studied in various animal models, revealing a wide array of mechanisms through which cocoa-derived compounds modulate inflammatory responses. These studies provide crucial insights into the potential therapeutic applications of cocoa extracts in inflammatory conditions. Cocoa polyphenols demonstrate significant anti-inflammatory activity through multiple pathways. In ICR mice, cocoa polyphenols have been shown to lower the expression of cyclooxygenase-2 (COX-2), a key enzyme in the inflammatory process. Furthermore, they inhibit the nuclear translocation of p65, a subunit of NF-κB, and prevent the degradation of IκBα, thus suppressing the NF-κB signaling pathway, which is central to inflammatory responses [81]. Studies in C57BL/6 mice have revealed that cocoa supplementation decreases the mRNA levels of pro-inflammatory cytokines such as TNF-α and IL-6, as well as nitric oxide synthase. Additionally, cocoa extracts inhibit the activity of enzymes associated with inflammation, including phospholipase A2 and cyclooxygenase-2 [82]. These effects collectively contribute to a comprehensive anti-inflammatory action. The anti-inflammatory properties of cocoa extracts extend to wound healing. In rabbits, cocoa pod extract has been shown to accelerate wound healing, likely due to its rich content of polyphenols, flavonoids, and tannins [83]. This suggests potential applications in dermatological conditions and post-surgical recovery. Cocoa extracts also demonstrate significant metabolic effects that indirectly contribute to their anti-inflammatory action. In Wistar rats, a diet enriched with cocoa bean phenolic extract led to a decrease in fat tissue, inhibition of protein tyrosine phosphatase 1B (PTP1B), attenuation of hepatic steatosis, and improved serum lipid profiles [84]. These metabolic improvements can reduce systemic inflammation associated with obesity and metabolic disorders. The immunomodulatory effects of cocoa are further evidenced by studies in Wistar rats, where cocoa-enriched diets caused a decrease in prostaglandin E2 (PGE2) production and prevented imbalances in T-cell proportions [85]. This suggests that cocoa compounds can modulate both innate and adaptive immune responses. Cocoa extracts have also shown promise in specific inflammatory conditions. In a rat model of periodontitis, cocoa pod husk extract increased alveolar bone regeneration by enhancing osteoblast numbers and bone morphogenetic protein-2 (BMP-2) expression [86]. This indicates potential applications in inflammatory bone disorders. Ethanol extracts of cocoa stem bark have been shown to reduce inflammation in Wistar rats by lowering neutrophil migration and reducing inflammatory mediator production [85]. Cocoa extracts also demonstrate antioxidant properties that complement their anti-inflammatory effects. Polyphenol-enriched cocoa leaf extracts inhibit xanthine oxidase and angiotensin-converting enzymes in male Wistar rats, indicating potential in preventing oxidative stress [87]. Finally, dietary studies in female Wistar rats have shown that cocoa-containing diets lower serum levels of TNF-α and inducible nitric oxide synthase (iNOS) activity, while also decreasing colon cell infiltration [88]. This suggests potential applications in inflammatory bowel conditions. In conclusion, the in vivo anti-inflammatory effects of Theobroma cacao extracts are mediated through multiple mechanisms, including the modulation of inflammatory enzymes, suppression of pro-inflammatory cytokines, inhibition of NF-κB signaling, improvement of metabolic parameters, and enhancement of wound healing. These diverse effects underscore the potential of cocoa-derived compounds as novel anti-inflammatory agents with wide-ranging therapeutic applications. However, further research, including clinical trials, is necessary to fully elucidate the efficacy and safety of these extracts in human inflammatory conditions. Other studies are presented in Table 4 below.

While numerous in vitro and in vivo studies have demonstrated the anticancer and anti-inflammatory potential of Theobroma cacao, clinical evidence has been more limited. However, several important clinical trials have specifically investigated these properties in human subjects.

A particularly significant clinical investigation examining the anticancer effects of Theobroma cacao is the “Effects of Chocolate Consumption in Elderly Patients With Cancer in Palliative Care” trial (NCT04367493). This randomized intervention study specifically focused on elderly cancer patients receiving palliative care, providing direct clinical evidence of cocoa’s effects in cancer patients. The study design involved three groups of 15 subjects each: a control group, a 55% cocoa intervention group, and a white chocolate intervention group. The trial measured multiple key parameters, including inflammatory markers through serum interleukin-6 levels, oxidative stress parameters via reduced glutathione (GSH) levels and ascorbic acid quantification, lipid peroxidation through malonaldehyde measurements, and DNA damage assessment using 8-hydroxy-2′-deoxyguanosine levels. Additionally, the study evaluated quality of life measures using the EORTC-QLQ-C30 questionnaire.

The COcoa Supplement and Multivitamin Outcomes Study (COSMOS; NCT02422745) represents a large-scale investigation into cocoa’s potential anticancer properties. This randomized clinical trial examined whether daily supplementation with cocoa extract (containing 500 mg/day of flavanols, including 80 mg of (−)-epicatechin) could reduce cancer risk in older adults. The scale and design of this study provide valuable insights into the potential role of cocoa flavanols in cancer prevention.

An ancillary study to COSMOS (NCT05510375) specifically investigated the anti-inflammatory aspects of cocoa supplementation through the examination of inflammaging—the chronic, low-grade inflammation associated with aging. This study is particularly relevant, as it bridges the gap between laboratory findings on cocoa’s anti-inflammatory properties and their clinical manifestation in human subjects.

These clinical trials represent critical steps in validating the anticancer and anti-inflammatory properties of Theobroma cacao observed in preclinical studies. They provide important insights into the translation of laboratory findings to human applications and suggest potential therapeutic roles for cocoa compounds in cancer care and inflammation management.

## 6. Antitumor and Anti-Inflammatory Effects of *Theobroma cacao* Extract in an In Vitro and In Vivo Model in Combination with Nanoparticles

Nanoparticles have emerged as a promising platform for enhancing cancer therapy due to their unique physicochemical properties and ability to be functionalized with various targeting moieties and therapeutic agents. Their small size allows them to penetrate biological barriers and accumulate preferentially in tumor tissues through the enhanced permeability and retention (EPR) effect [91,92]. Nanoparticles can be engineered to carry multiple drugs simultaneously, enabling combination therapies and overcoming drug resistance mechanisms. Moreover, stimuli-responsive nanoparticles can be designed to release their payload in response to specific tumor microenvironment conditions, such as pH or enzyme activity, thereby improving the therapeutic index and reducing systemic toxicity. The surface of nanoparticles can also be modified with imaging agents, allowing for theranostic applications that combine therapy and diagnostics [93,94,95].

In the context of inflammatory disorders, nanoparticles offer several advantages for targeted drug delivery and the modulation of the inflammatory response. Their ability to encapsulate and protect anti-inflammatory compounds can enhance drug stability and prolong circulation time. Nanoparticles can be designed to target specific cells or tissues involved in inflammation, such as activated endothelial cells or macrophages, through the incorporation of appropriate ligands. This targeted approach can improve the efficacy of anti-inflammatory treatments while minimizing off-target effects [96,97,98]. Additionally, certain types of nanoparticles, such as metal nanoparticles, have inherent anti-inflammatory properties that can complement the effects of loaded drugs. The versatility of nanoparticle platforms allows for the development of multifunctional systems that can simultaneously deliver therapeutics, modulate the immune response, and provide real-time monitoring of treatment efficacy [99,100].

The literature is limited in relation to biological studies on the anticancer and anti-inflammatory effects of *Theobroma cacao* extract in combination with nanoparticles, which is why further work confirming this synergistic effect is so important. Table 5 below shows current studies that demonstrate combinations of *Theobroma cacao* extract in combination with nanoparticles in the context of biological research.

## 7. Conclusions and Future Perspectives

In conclusion, *Theobroma cacao* appears to offer complex and promising therapeutic potential based on its anti-inflammatory and anticancer properties, which can be attributed to its polyphenol, flavonoid, or alkaloid content. The extracts have demonstrated significant antiproliferative, proapoptotic and potentially antimetastatic properties in various cancer cell lines and animal models, both alone and in combination with nanoparticles. An important aspect of *T. cacao* extracts is that they are able to demonstrate potential synergistic effects with conventional chemotherapeutic agents and protect against chemotherapy-induced organ toxicity. This dual action suggests a unique role for cacao-derived compounds in enhancing the efficacy of cancer treatment, while mitigating its side effects.

**Figure 4:**
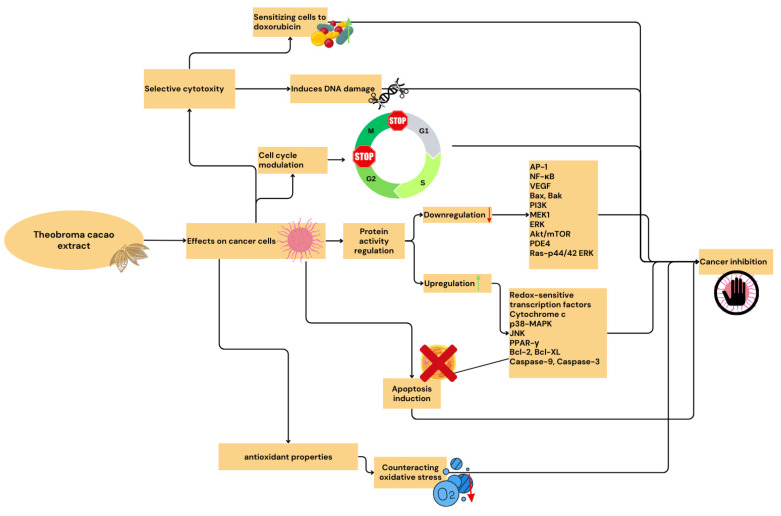
The potential mechanism of action of cocoa extract (Canva, version 1.97.0.0, Canva Inc., Sydney, Australia, https://www.canva.com accessed on 26 June 2024).

Further research into combination therapies, particularly the synergistic effects between *Theobroma cacao* extracts and conventional treatments, may open up new avenues in cancer therapy and the treatment of chronic inflammatory diseases. Although several mechanisms of action have been identified, a deeper understanding of the molecular pathways involved in the anticancer and anti-inflammatory effects of *Theobroma cacao* extracts is needed. The integration of nanotechnology with *T. cacao* extracts represents a particularly promising direction for future research. Nanoparticle-based delivery systems offer several advantages, including enhanced bioavailability, targeted delivery, and controlled release of bioactive compounds. Future studies should focus on optimizing nanoformulations, investigating their biodistribution and safety profiles, and evaluating their long-term efficacy in various therapeutic applications. The development of new formulations and delivery systems may enhance the bioavailability and targeted delivery of bioactive cacao compounds, potentially improving their therapeutic efficacy. Long-term research into the effects of cocoa consumption on the prevention of cancer and chronic inflammation is warranted, as is research into how individual genetic variants may influence the response to *Theobroma cacao* extracts, potentially paving the way for personalized therapeutic strategies. The potential mechanism of action of *Theobroma cocoa* extract is shown in Figure 4.

In summary, *Theobroma cacao* has long played a key role in the global economy. Recent research shows that it can play a key role in health protection. The economic importance of cocoa is obvious in the food industry, and its medicinal properties, especially its anti-inflammatory and anticancer properties, offer promising opportunities for health. Continuous research and development in the cultivation and practical use of cocoa may further increase its share in both the global economy and biomedicine in the future. Its multifaceted biological activities, combined with a long history of human consumption and enhanced effects when combined with nanoparticles, make it an attractive candidate for further research.

## Figures and Tables

**Table 3:** In vivo anticancer effect of *Theobroma cacao* extract.

Part of the Plant	Class of Compounds	Organism	Dose	N	Exposure Time	Activity/Mechanism/Effects	Ref.
Cocoa seeds glycolic extract	polyphenols	Mice bone marrow	Genotoxicity test: 0.5, 1.0, 1.5, and 2.0 g/kg of theobroma extract Antigenotoxicity test: 2.0 g/kg of theobroma extract and 5.0 mg/kg of doxorubicin	5 male mice and 5 female mice	24 h 48 h	The combination of cocoa extract (2 g/kg) with doxorubicin (5 mg/kg) partially increases systemic toxicity. The extract enhances the toxic effects of doxorubicin.	[77]
Cocoa cake extract	polyphenols, flavonoids, gallic acid, procyanidin B1, epigallocatechin, catechin, procyanidin B2, epicatechin, epigallocatechin gallate, vanillin, p-coumaric acid, m-coumaric acid, quercetin	BALB/cN mice liver and blood samples	Cocoa extract—34.5 mg/kg Epicatechin extract—2.24 mg/kg	4 groups of 8 female BALB/cN mice	2 weeks	Cocoa polyphenols improved cellular redox state and molecular signaling pathways associated with oxidative stress. They also decreased hepatic cell necrosis and suppressed Pkm2. Hepatotoxicity was decreased by lowering the activity of serum transaminases and phosphatic phosphatase activity. The extract acted as a carcinogenic inhibitor by down-regulating Hsp 90 and returning the expression of Hsp70 to normal levels.	[78]
Cocoa nibs powder	polyphenols, alkaloids, flavonoids	Female BALB/c mice vital organs (heart, liver, kidney) and blood samples	Doxorubicin-treated mice—4.91 mg/kg Cocoa extract-treated mice—200 mg/kg Doxorubicin- and cocoa-treated mice—4.91 mg/kg of doxorubicin and 200 mg/kg of cocoa extract	5 groups of 16 mice	21 days	Cocoa extract protects organs against doxorubicin-induced intoxication (heart, liver, kidney). It also has a synergistic anticancer effect, enhancing doxorubicin activity.	[79]
Acticoa powder	flavonoids, flavonol, epicatechin, procyanidins	Wistar–Unilever rat prostate	Acticoa powder 24 mg/kg, Acticoa powder 48 mg/kg	4 groups of 15 rats	9 months	Daily consumption of cocoa products influence plasma flavonol concentration, enhancing the antioxidant potential of plasma. Treatment with Acticoa powder protects rats from CI prostate tumor.	[80]
Cocoa pods powder	polyphenols, flavonoids, alkaloids	BALB/c mice Ehrlich ascites carcinoma tumor cells	Doxorubicin-treated mice—4.91 mg/kg Cocoa extract-treated mice—200 mg/kg Doxorubicin and cocoa-treated mice—4.91 mg/kg of doxorubicin and 200 mg/kg of cocoa extract Pretreated mice—200 mg/kg of cocoa extract for 21 days before cancer induction. After cancer induction, the mice received 200 mg/kg of cocoa extract and 4.91 mg/kg of doxorubicin	6 groups of 10 mice	21 days	Cocoa powder protects against vital organ damage induced by doxorubicin without compromising chemotherapeutic effects. Moreover, it can neutralize free radicals.	[17]

**Table 4:** In vivo anti-inflammatory effect of *Theobroma cacao* extract.

Part of the Plant	Class of Compounds	Organism	Dose	N	Exposure Time	Activity/Mechanism/Effects	Ref.
Cocoa polyphenols from cocoa powder	Polyphenols, Gallic acid, Epicatechin.	Skin from ears of ICR mice	With 12-O-tetradecanoylphorbol-13-acetate: 4 mg/kg 20 mg/kg 40 mg/kg 200 mg/kg Without 12-O-tetradecanoylphorbol-13-acetate: 200 mg/kg	Groups of 6 female mice	5 h	Cocoa polyphenols lower the activity of COX-2 expression, inhibit the activation of MAPK and NF-κB pathways.	[84]
Unsweetened cocoa powder	Polyphenols.	Male C57BL/6 mice	80 mg/g of unsweetened cocoa powder	Low-fat diet 23 mice High-fat diet 21 mice High-fat diet cocoa treated 24 mice	18 weeks	Cocoa supplementation decreases adipose tissue inflammation by downregulating genes associated with NF-κB.	[82]
Cocoa pod extract	Polyphenols, Flavonoids, Tannins.	Rabbit	Concentration of cocoa: 0%, 5%, 10%, 15%	36 male rabbits	3 days 5 days 7 days	Cocoa pod extract can accelerate the speed of wound healing.	[83]
Cocoa bean phenolic extract	Phenols	Wistar rats	Raw cocoa bean: 2.25% of diet; Roasted cocoa bean: 2.45% of diet; Flavan-3-ol: 0.114% of diet	5 groups of 8 male rats	4 weeks	A diet enriched with cocoa bean extract causes fat tissue reduction, PTP1B inhibition, hepatic steatosis attenuation, ROS protection, and improved serum lipid profile, and increases serum ACl. It shows anti-obesity properties.	[89]
Leaf extracts	Tannins, Phenols, Saponins, Terpenoids, Flavonoids, Glycosides.	African earthworms (*Pheretima posthuma*)	10, 25, 50 mg/mL	-	-	Cocoa leaf extracts have antioxidant and anthelmintic activity.	[90]
Cocoa pods powder	Flavonoids, Alkaloids, Tannins, Saponins.	Wistar rats	Fermented cocoa: 150 mg/kg 300 mg/kg	35 rats	21 days	Anti-hyperglycemia compounds were discovered in fermented cocoa polyphenol extracts.	[85]
Cocoa pod husk extract	Polyphenols	Wistar rats	Cocoa pod husk extract 100 mg/mL	24 male rats	7 days 14 days	Cocoa pod husk extract increases alveolar bone regeneration in periodontitis by increasing osteoblast numbers and BMP-2 expression.	[86]
Stem bark extract	-	Wistar rats	Cocoa stem bark: Extract 250 mg/kg Ethylacetate fraction 65, 125 and 250 mg/kg	7 groups of 10 rats	72 h	Ethanol extracts of cocoa stem bark reduce inflammation by decreasing inflammatory mediator production (TNF-α).	[81]
Cocoa leaves	Polyphenols	Male Wistar rats	10, 20, 30, 40 μg/mL	3 male rats	7 days acclimatization	Polyphenol-enriched cocoa leaf extract inhibits xanthine oxidase and angiotensin 1-converting enzyme. Leaf extract may be useful in preventing oxidative stress.	[87]
Cocoa	Procyanidin B2 Catechin, Epicatechin, Isoquercetin, Quercetin	Female Wistar rats	5% cocoa diet	4 groups of 12 rats	3 weeks	Cocoa-containing diet shows anti-inflammatory potential. In addition, lower colon cell infiltration was observed.	[88]

**Table 5:** Biological effect of nanoparticles with *Theobroma cacao* extract.

Part of the Plant	Nanoparticles	Dose	Activity/Mechanism/Effects	Ref.
Cocoa powder	Silver nanoparticles	25, 50 mg/mL	An insignificant cytotoxic effect on human dermal fibroblast cells has been demonstrated.	[101]
Cocoa seeds	Palladium/Copper (II) oxide nanoparticles	15 mL of cocoa seed extract	The chemical components of cocoa (e.g., catechin, phenolic acids) were identified as stabilizing, reducing and capping agents. This method of obtaining nanoparticles shows lower toxicity and is environmentally friendly.	[102]
Cocoa powder	Gold nanoparticles	0.1, 1, 2.5, 10, 50 mg/mL	Gold nanoparticles are not toxic for human dermal fibroblast.	[103]
Cocoa seed extract	Gold nanoparticles	200, 250, 270, 300 μL	Anisotropic b-AuNPs (derived from *T. cacao* [cocoa] seed extract) demonstrated excellent photothermal properties in A431 epidermal cancer cells using a laser power density of 6 W/cm^2^. The b-AuNP nanoparticles exhibited near-infrared absorbance at 700–1000 nm, facilitating an effective photothermal therapy against A431 cancer cells.	[104]
Cocoa bean extract	Graphene nanoparticles	IC_50_—31.2 µg/mL	The cytotoxicity studies showed that the synthesized CSE-GQDs exhibited dose-dependent toxicity on human breast cancer (MCF-7) cell lines.	[105]

## Data Availability

Data will be made available on request.

## Abbreviations

GDP	Gross domestic product
ALT	Alanine transaminase
BDNF	Brain-derived neurotrophic factor
HRS	Hodgkin and Reed-Sternberg
THP-1	Human acute monocytic leukemia
TAMs	Tumor-associated macrophages
ROS	Reactive oxygen species
MMP2	Matrix mettaloproteinase-2
MAPK	Mitogen activated protein kinases
NO	Nitric oxide
TNF	Tumor necrosis factor
COX-2	Cyclo-oxygenase-2
PTP1B	Protein tyrosine phosphatase 1B
PGE2	Prostaglandin E2
BMP-2	Bone morphogenetic protein-2
EPR	Permeability and retention effect
